# P-34. Patients’ Survey of Human Papilloma Virus Vaccination among Systemic Lupus Erythematosus Patients in Japan after Suspension of Proactive Recommendation

**DOI:** 10.1093/ofid/ofae631.241

**Published:** 2025-01-29

**Authors:** Takashi Kurita, Akio Yamamoto, Tadashi Hosoya, Marina Tsuchida, Shinsuke Yasuda, Yoshiaki Gu

**Affiliations:** Graduate School of Medical and Dental Sciences, Tokyo Medical and Dental University (TMDU), Bunkyo-ku, Tokyo, Japan; Graduate School of Medical and Dental Sciences, Tokyo Medical and Dental University (TMDU), Tokyo, Bunkyo-ku, Tokyo, Japan; Graduate School of Medical and Dental Sciences, Tokyo Medical and Dental University (TMDU), Bunkyo-ku, Tokyo, Japan; Graduate School of Medical and Dental Sciences, Tokyo Medical and Dental University (TMDU), Bunkyo-ku, Tokyo, Japan; Graduate School of Medical and Dental Sciences, Tokyo Medical and Dental University (TMDU), Bunkyo-ku, Tokyo, Japan; Tokyo Medical and Dental University, Bunkyo-ku, Tokyo, Japan

## Abstract

**Background:**

Systemic lupus erythematosus (SLE) patients are known to have a high incidence of cervical cancer. The main reason for this is considered to reflect the high rate of Human papilloma virus (HPV) infection and inadequate HPV clearance due to immunosuppressive status. Japan has been facing a problem of low HPV vaccination rates due to the suspension of proactive recommendations for routine HPV vaccination for more than eight years because of the negative media campaign. The objective of this study is to identify the factors that inhibit HPV vaccination among SLE patients to develop strategies for improving HPV vaccination rate.

Figure

**Figure:**
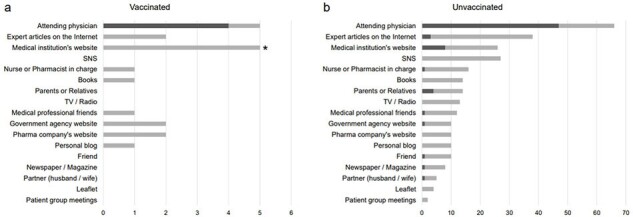

Sources of medical information for HPV vaccinated and unvaccinated participants. We asked which informational tools participants use to gather medical information. The most trusted sources are indicated by dark bars; others are indicated by light bars.

* indicates significant differences between HPV vaccinated and unvaccinated participants (p<0.05, chi-square test). (a): Answers by HPV vaccinated participants. (b): Answers by HPV unvaccinated participants.

HPV, Human papilloma virus; SNS, Social networking service; TV, Television

**Methods:**

We conducted a questionnaire survey of female SLE patients aged 18-45 years attending our hospital to analyze factors related to HPV vaccination.

**Results:**

We obtained responses of 83 participants. Only 5 (6.0%) were received HPV vaccination, 15 (18.1%) were uncertain of their vaccine history, and 27 (32.5%) had never even heard of HPV vaccination. The reasons for unvaccinated against HPV were "don't know" with 24 participants, "missed opportunity" with 15, and "troublesome, somehow" with 8. The most trusted source of medical information for the unvaccinated was their physician (47, 60.3%) (Figure). Among the unvaccinated, those who wished to be vaccinated in the future were positively correlated with “trust of vaccine benefit” (r=0.515. p=0.014) and “accurate knowledge about HPV vaccine” (r=0.614, p=0.002), and negatively correlated with “negative attitudes toward vaccination and vaccine policy” (r=-0.562, p=0.007).

**Conclusion:**

HPV vaccination rate among SLE patients in Japan was extremely low. The main reason was lack of knowledge. The most effective solution is considered to provide accurate information and adequate recommendations of HPV vaccination by attending physicians.

**Disclosures:**

**Yoshiaki Gu, MD, PhD, MPH**, MSD K.K.: Grant/Research Support

